# Encapsulation of Bacteriophage in Liposome Accentuates Its Entry in to Macrophage and Shields It from Neutralizing Antibodies

**DOI:** 10.1371/journal.pone.0153777

**Published:** 2016-04-26

**Authors:** Saloni Singla, Kusum Harjai, Om Prakash Katare, Sanjay Chhibber

**Affiliations:** 1 Department of Microbiology, Panjab University, Chandigarh, India; 2 University Institute of Pharmaceutical Science, Panjab University, Chandigarh, India; Queen's University Belfast, UNITED KINGDOM

## Abstract

Phage therapy has been a centre of attraction for biomedical scientists to treat infections caused by drug resistant strains. However, ability of phage to act only on extracellular bacteria and probability of interference by anti-phage antibodies *in vivo* is considered as a important limitation of bacteriophage therapy. To overcome these hurdles, liposome were used as delivery vehicle for phage in this study. Anti-phage antibodies were raised in mice and pooled serum was evaluated for its ability to neutralize free and liposome entrapped phage. Further, ability of phage and liposome-entrapped phage to enter mouse peritoneal macrophages and kill intracellular *Klebsiella pneumoniae* was compared. Also, an attempt to compare the efficacy of free phage and liposome entrapped phage, alone or in conjunction with amikacin in eradicating mature biofilm was made. The entrapment of phage in liposome provided 100% protection to phage from neutralizing antibody. On the contrary un-entrapped phage got neutralized within 3 h of its interaction with antibody. Compared to the inability of free phage to enter macrophages, the liposome were able to deliver entrapped phage inside macrophages and cause 94.6% killing of intracellular *K*. *pneumoniae*. Liposome entrapped phage showed synergistic activity along with amikacin to eradicate mature biofilm of *K*. *pneumoniae*. Our study reinforces the growing interest in using phage therapy as a means of targeting multidrug resistant bacterial infections as liposome entrapment of phage makes them highly effective *in vitro* as well as *in vivo* by overcoming the majority of the hurdles related to clinical use of phage.

## Introduction

*Klebsiella pneumoniae* is an important pathogen that accounts for up to 8% of nosocomial infections in the Western world. It is placed among the eight most infectious agents in hospitals [1]. Its incidence is often linked to use of artificial devices, indicating that bacterial adhesion and biofilm formation are important virulence attributes in the establishment of infection.

Lytic bacteriophage with antibacterial property has been recommended as an adjunct therapy to antibiotics as it offers a number of advantages over the existing chemical agents. However, production of neutralizing antibodies, emergence of phage-resistant bacterial strains and restricted intracellular entry of phage are considered as major hurdles in the clinical use of phage [2–5]. The production of anti-phage antibodies has been reported following phage therapy [6,7]. These antibodies have the ability to inactivate the phage due to their neutralizing nature. However, the actual impact of these antibodies on the efficacy of repeated phage dosing is unclear.

Previous researchers have suggested that bacteriophages can be used to treat bacterial infections as long as bacteria remain accessible. However, it has also been suggested that phage therapy is not suitable for intracellular bacteria because of their inability to enter into myeloid cells. *K*. *pneumoniae* was initially considered as an extracellular pathogen but now it is clear that it is also capable of intracellular survival in a variety of cells including phagocytic cells (macrophages) and epithelial cells [8,9,10,11]. Survival of *K*. *pneumoniae* within epithelial cells may serve as a critical reservoir from where reinfection of the host can take place. Most of the antibiotics show poor penetration within eukaryotic cells, leading to persistence of pathogens.

Recently, we have reported that entrapment of phage in liposome could be considered as a powerful tool against *K*. *pneumoniae* induced respiratory tract infection in mice as it resulted in mounting a highly effective therapeutic and prophylactic response *in vivo* [12]. The present study was designed to ascertain the reason for the higher efficacy of liposome entrapped phage as compared to free phage.

In the present study, liposome were used as a delivery vehicle for the already characterized lytic phage KPO1K2 and this liposome entrapped phage alone or in conjunction with antibiotic was evaluated for its ability to eradicate mature biofilm of *K*. *pneumoniae*. In addition, ability of liposome to protect phage from neutralizing antibodies and to carry it into phagocytic compartment to kill engulfed *K*. *pneumoniae* persisting within murine macrophages was also attempted using *ex vivo* experiments.

## Materials and Methods

### Ethics Statement

The experimental protocols were approved by the Institutional Animal Ethics Committee of Panjab University, Chandigarh, India (Approval ID: IAEC/156). Animal experimentation was performed in accordance with the guidelines of Committee for the Purpose of Control and Supervision of Experiments on Animals (CPCSEA), Government of India. All efforts were made to minimize the suffering of animals.

### Bacterial strain, bacteriophage and antibiotic

*Klebsiella pneumoniae* B5055 (MTCC 5832) obtained originally from Dr M. Trautman, Department of Medical Microbiology and Hygiene, University of Ulm, Ulm, Germany and maintained in the laboratory was used in the present study. This strain expresses O1 and K2 antigens, which makes it the most commonly encountered serotype in clinical situations. All experiments were performed in nutrient broth medium. Growth media and antibiotic were purchased from HiMedia Laboratories, Mumbai, India. Stock solution of amikacin was prepared according to the method of Andrews [13]. A depolymerase producing lytic bacteriophage (KPO1K2) (MTCC 5831), already characterized in our laboratory was used in the present study [14]. The phage belongs to family Podoviridae with a designation of T7-like lytic bacteriophage. KPO1K2 possessed icosahedral head with pentagonal nature with apex to apex head diameter of about 39 nm and short non-contractile tail (10 nm). The ability of the bacteriophage to produce depolymerase enzyme, broad host range (able to infect some clinical isolates of *K*. *pneumoniae*), stability over a wide range of pH (4–11) and a small latent period and large burst size make it very useful to use clinically [14]. All bacteriophage and bacterial dilutions were made in sterile normal saline.

### Production of phage

High titre of bacteriophages was prepared by standard protocol of Hughes *et al*. [15]. A single colony of *K*. *pneumoniae* B5055 was inoculated in 10 ml of nutrient broth and incubated at 37°C overnight. 5.0 ml of overnight culture was transferred to a 30 ml glass tube to which 100 μl of bacteriophage suspension (10^8^ PFU/ml) was added. Mixture was incubated at 37°C for 20 min to allow adsorption of bacteriophage onto the bacteria. 4.0–5.0 ml of prewarmed nutrient broth (37°C) was then added to the tube and reincubated at 37°C under shaking conditions until complete lysis occurred. After complete lysis, 100 μl of chloroform was added, vortexed and centrifuged at 10,000 X g for 10 min at 4°C. Supernatant was transferred to a sterile tube after passing through 0.45 μm membrane and stored at 4°C. Filtrate collected was used as a source of bacteriophages. Phage titration was performed according to soft agar overlay technique described by Adam [16].

### Preparation of Liposome entrapped bacteriophage

**Fig 1:**
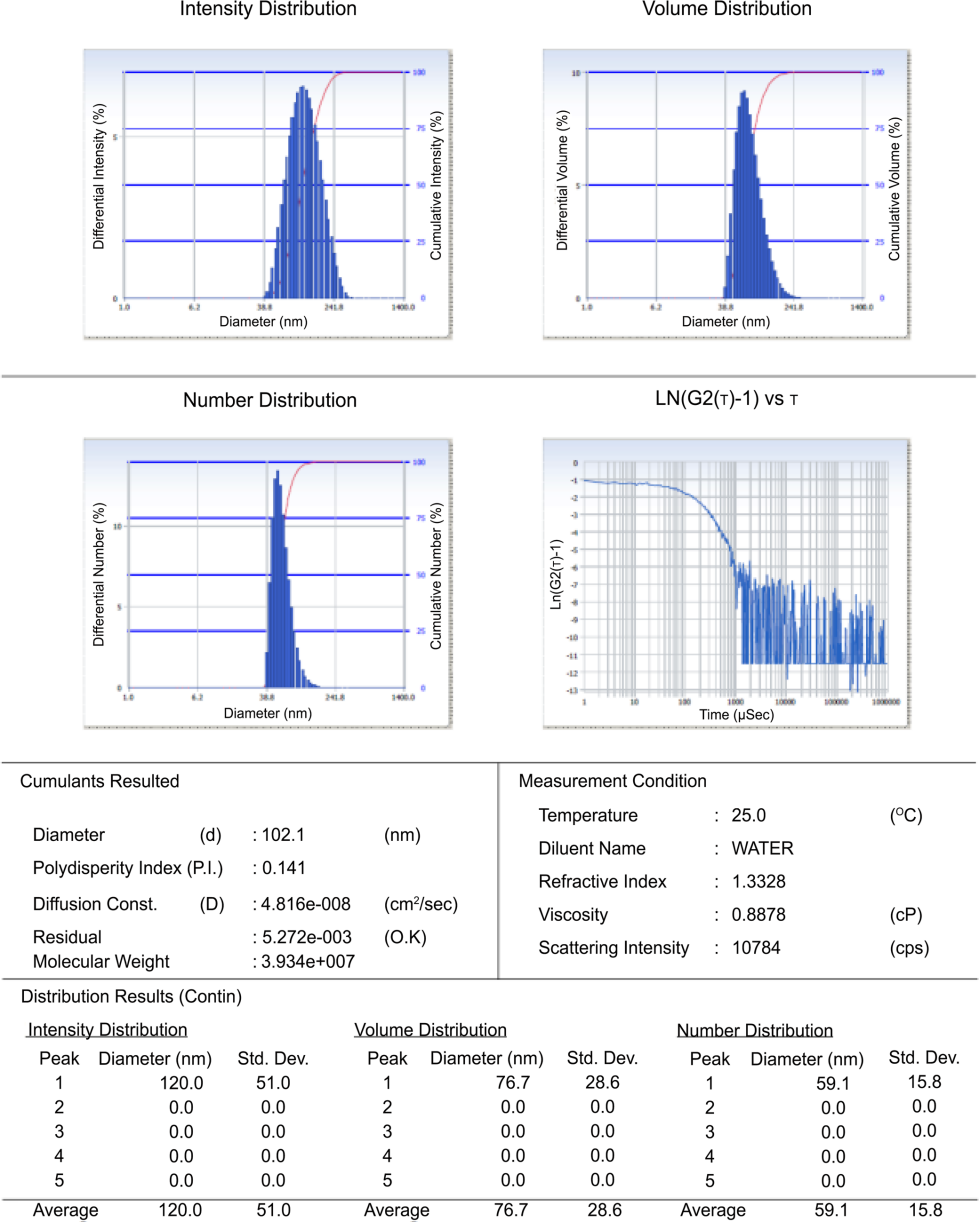
Particle size distribution and PDI of liposomal formulation of phage.

Liposomal formulations were developed using well-established rota-evaporation technique [17]. Cationic liposomal formulation of bacteriophage was prepared using mass ratio (45mg/3ml formulation) of Phosphatidyl choline: Cholesterol: Tween 80: Stearylamine (PC:CHOL:T-80: SA) as 9:1:2:0.5 by thin film technique using hydration temperature of 40°C. Suspension containing bacteriophage was used as hydration media. Cationic liposomal formulation of phage was used in the present study for *in vitro*, *in vivo* and *ex vivo* experiments. The average particle size of liposome entrapped phage was found to be 102.1 nm and poly dispersity index (PDI) value of the system was 0.141 as shown in Fig 1.

Number of liposomes was determined by the method of Lasic DD [18]. Formula used to determine the number of liposomes was

$$N=\frac{{\text{M}}_{\text{lipid}}\times {\text{N}}_{\text{A}}}{{\text{N}}_{\text{total}}\times 1000}\;\text{where}\;N_{\mathrm{total}}=\frac{8\pi r^{2}}{\text{a}}$$

M_lipid_ is the molar concentration of lipid used

N_A_ is the Avogadro number and it is equal to 6.02 X 1023

N_total_ is the total number of lipids per liposome

r is the radius of liposome

And a is the average area of lipid head groups

Same amount of lipid/ml and similar conditions were used to prepare empty liposomes and liposome entrapped phage. So, we had same number of liposomes in both the preparations i.e. 6 x 10^13^/ml (r = 51 nm, a = 0.679 nm^2^, M_lipid_: 9.35 mM).

### Biofilm development

The method of Bedi *et al*. [19] originally derived from Christensen [20] was used to establish *K*. *pneumoniae* biofilm in 96-well microtiter plate. Briefly, wells of microtiter plate were inoculated with 0.1 ml of nutrient broth medium and 0.1 ml of *K*. *pneumoniae* culture (10^8^ CFU/ml)and incubated at 37°C overnight. In each test, plate sterility control was included that contained sterile nutrient broth. After incubation, planktonic cells in fluid were removed and wells washed thoroughly 3 times with NS. Sterile pipette tip was then used to scrap the biofilm matrix. Biofilm matrix was suspended in NS and vortexed for 3 min. Viable cell count method was used to enumerate microbial load. In rest of the wells, spent medium was replaced with fresh sterile nutrient broth and plate reincubated at 37°C overnight. Media in each well was replaced every 24 h till 7^th^ day of experiment.

### Biofilm susceptibility assay

Biofilm grown for different time intervals were exposed to 200 μl of amikacin (40 μg/ml) and 200 μl of bacteriophage at multiplicity of infection (MOI) of 1.0, alone or in combination. Amikacin at 40 μg/ml was used as highest efficacy was seen at this concentration out of all the concentrations of this antibiotic tested [21]. Also, biofilm on a microtiter plate was exposed to liposome entrapped phage alone or in combination with amikacin (40 μg/ml). As a control, biofilm of different days was exposed to plain liposome. Number of liposomes present in the plain liposome preparation and liposomal phage was 1.2 X 10^13^ in 200 μl that was added to treated wells. Each experiment was carried out in duplicate, so two wells were harvested each day per treatment.

Untreated biofilm was washed three times with normal saline and bacterial numbers in the biofilm of all ages were estimated. Treated biofilm was washed three times with normal saline before and after each treatment. Viable cell count of the scraped biofilm matrix was estimated by plating the appropriate dilution of bacterial culture and reported as log_10_ reduction value [22].The concentration of bacteriophage added to each well was adjusted as per the bacterial count of untreated biofilm on different days, keeping the MOI constant at 1.

### Animals

Female BALB/c mice, 6–8 weeks old (20–25 g) were procured from the central animal house of Panjab University, Chandigarh, India. The animal experiment protocols were duly approved by the *Institutional Animal Ethics Committee* (Approval ID: IAEC/156), Panjab University, Chandigarh, India. Animals were housed in accordance with the guidelines of Committee for Purpose of Control and Supervision of Experiments on Animals (CPCSEA), Government of India. Food and water was provided ad libitum. All possible efforts were made to decrease the suffering of animals.

#### Raising antibodies against phage

Method described by Capparelli *et al*. [23] with some modifications was used to raise antibodies against bacteriophage KPO1K2 in BALB/c mice. Each mouse received intraperitoneal (i.p.) injection of 10^9^ PFU/ml emulsified with an equal volume of complete Freund’s adjuvant. After 2 weeks, booster dose consisting of 10^9^ PFU/ml emulsified with an equal volume of incomplete Freund’s adjuvant was given i.p. The mice were bled after 1 week of booster dose. The source of antibodies against phage was the serum of a mouse. Mouse serum was stored at 0°C till use.

### Phage neutralization test

100 μl of phage (10^6^ PFU/ml) was incubated with 100 μl ml of serum having antibodies against phage (dilutions 1X 10^−1^ and 1X 10^−2^) for 3 h at room temperature. 100 μl of liposome entrapped phage (10^6^ PFU/ml) was also incubated with 100 μl of serum having antibodies against phage (1X 10^−1^) for 3 h at room temperature. As a control, 100 μl of phage (10^6^ PFU/ml) was incubated with 100 μl of normal mouse serum (neat) for 3 h at room temperature. Bacteriophage titre (PFU/ml) was calculated for all test and control samples at different time intervals by soft agar overlay method [23]. At the end of incubation 1ml of bacteria (10^5^ CFU/ml) in tryptic soy broth was added to all reaction mixtures and further incubated for 3 h at 37°C. After 3 h, cell count was determined by plating appropriate dilution on nutrient agar and phage titre was estimated by the soft agar overlay method [16].

### Isolation and culturing of mouse peritoneal macrophages

Five female BALB/c mice were injected with 3% thioglycollate broth (HiMedia, Mumbai, India) and mice were euthanized by cervical dislocation after 4 days. The intact peritoneal wall was exposed by manual retraction of the abdominal skin. 5 ml of ice cold Dulbeco’s modified Eagle medium (DMEM) (Sigma, St. Louis, MO, USA) supplemented with 10% fetal calf serum (FCS) (Gibco Invitrogen, Paisley, United Kingdom) was injected into peritoneal cavity. Gentle massaging of peritoneal cavity was done for 5 min. Fluid was aspirated from the cavity using the same syringe. The fluid collected from each individual mouse was pooled, centrifuged and washed thrice using ice cold phosphate buffered saline (PBS, pH 7.4). It was finally suspended in DMEM and kept on ice.

#### Viability of the macrophages

The viability of the macrophages was determined by counting cells in a haemocytometer using trypan blue (Sigma, St. Louis, MO, USA). 100 μL of cells were taken in a new eppendorf tube and added 400 μL 0.4% Trypan Blue (final concentration 0.08%) with gentle mixing. Counted the live, unstained cells (live cells do not take up trypan blue) in all 4 sets of 16 squares. Then, multiplied the average cell count from each of the sets of 16 corner squares by 10,000 (10^4^). Multiplied further by 5 to correct for the 1:5 dilution from the trypan blue addition. Viability was determined as number of viable macrophages/mL and count adjusted so as to achieve 1X10^5^ macrophages/well.

### Phagocytic uptake and killing

The ability of *K*. *pneumoniae* B5055 to resist phagocytic uptake and killing was assessed by the modified method of Capparelli *et al*. [23]. Modifications made in the original method included, usage of 10% FCS instead of 5% and addition of streptomycin to culture media only at first step to prevent contamination. Another modification was the use of different MOI of phage and bacteria due to difference in the bacteria used in two studies. Peritoneal mouse macrophage (1X10^5^/well) suspended in DMEM supplemented with FCS and streptomycin (25 μg/ml) was seeded in 12-well plate. Plate was then incubated overnight at 37°C in 5% CO_2_. Next day, cells were washed once with PBS and again suspended in fresh medium. *K*. *pneumoniae* cells (10^6^ CFU/well) were added. Plate was again incubated at 37°C in 5% CO_2_ to allow phagocytosis. 250 μl sample was withdrawn at appropriate time intervals and an equal volume of ice-cold PBS was added. Samples were then centrifuged at 1500 X *g* (Sigma Centrifuge) for 5 min. The supernatant was collected and pellets washed twice with 2 ml of ice-cold PBS. The pellet was resuspended in 20 μl of ice cold PBS having 0.5% Triton X solution and incubated at room temperature for 30 min. Viable intracellular bacteria and extracellular bacteria was determined in the treated pellet and supernatant respectively, by viable cell count method.

$$\%\;\text{uptake of bacteria}=\frac{{\text{N}}_{\text{T}}-{\text{N}}_{\text{E}}}{{\text{N}}_{\text{T}}}\text{X}\;100$$

$$\%\;\text{killing of intracellular bacteria}=\frac{({\text{N}}_{\text{T}}-{\text{N}}_{\text{E}})-N_{I}}{\left({\text{N}}_{\text{T}}-{\text{N}}_{\text{E}}\right)}\text{X}\;100$$

where N_T_ is the total number of bacteria added in each well

N_E_ in the number of extracellular bacteria (calculated in the supernatant)

N_I_ in the number of intracellular bacteria (calculated in the pellet)

## Comparison of intracellular killing activity of phage and liposome entrapped phage

Peritoneal mouse macrophages were distributed in 12-well plates (10^5^ cells/well) containing DMEM supplemented with 10% FCS and incubated overnight at 37°C in 5% CO_2_. Cells were washed once with PBS, resuspended in fresh medium and then infected with *K*. *pneumoniae* B5055 (10^6^ cells/well). The plates were then incubated for 2.5 h at 37°C in 5% CO_2_. Extracellular bacteria were killed with gentamicin (12.5 μg/well for 1 h). The cell suspension was later centrifuged at 1500 X *g* (Sigma Centrifuge) for 10 min at 4°C to remove the dead non-phagocytised bacteria and subsequent washings were given with DMEM. The cell pellet containing macrophages and engulfed bacteria was again re-suspended in 1 ml of DMEM containing 10% fetal calf serum. Cells were then exposed to phage (10^6^ PFU/well and 10^7^ CFU/well) at a MOI of 10 and 100 in duplicate. In addition other 4 wells containing macrophages were infected with liposome entrapped phage at a MOI of 10 and 100 in duplicate. The control wells were left without phage or liposome entrapped phage. The plate was then incubated for 24 h and samples withdrawn from all the wells at different time intervals i.e. 0 min, 3 h, 12 h and 24 h. Cells were lysed by adding Tween 20 (final concentration, 0.03%) to recover intracellular bacteria and each lysate was serially diluted in saline and plated to find viable bacteria. Cells were also stained with BacLight Live/Dead fluorescent stain (Molecular Probes, Eugene, OR, USA) after making smear on a glass slide and observed under confocal microscope. To show the specificity of Live/Dead bacterial stain, one control slide of heat killed bacteria was stained with Live/Dead stain. The ratio of live cells to dead cells was calculated by dividing the number of green cells (live cells) with the number of red cells (dead cells) using ImageJ version 1.46r.

### Statistical analysis

Results were analysed statistically by applying Student’s t-test for comparing efficacy of phage and liposomal phage on young and old biofilms, synergistic effect of phage and liposome entrapped phage with antibiotic on biofilm, and effect of neutralizing antibodies on phage and liposomal phage. Differences were considered statistically significant if p-values were less than 0.05. Change in CFU and PFU was calculated by the log reduction method [22].

## Results

### Antimicrobial treatment of biofilm grown in microtiter plate

#### Comparison of efficacy of phage and liposome entrapped phage on biofilm

Treatment of young biofilm (4 day old biofilm) using antibiotic (amikacin at 40 μg/ml) led to significant reduction of 2.0 logs in bacterial count (*P*<0.05).However, it was ineffective against the mature biofilm (5^th^ day onwards) as insignificant reduction of 0.6 in the log bacterial count (*P*>0.05) was seen.

**Fig 2:**
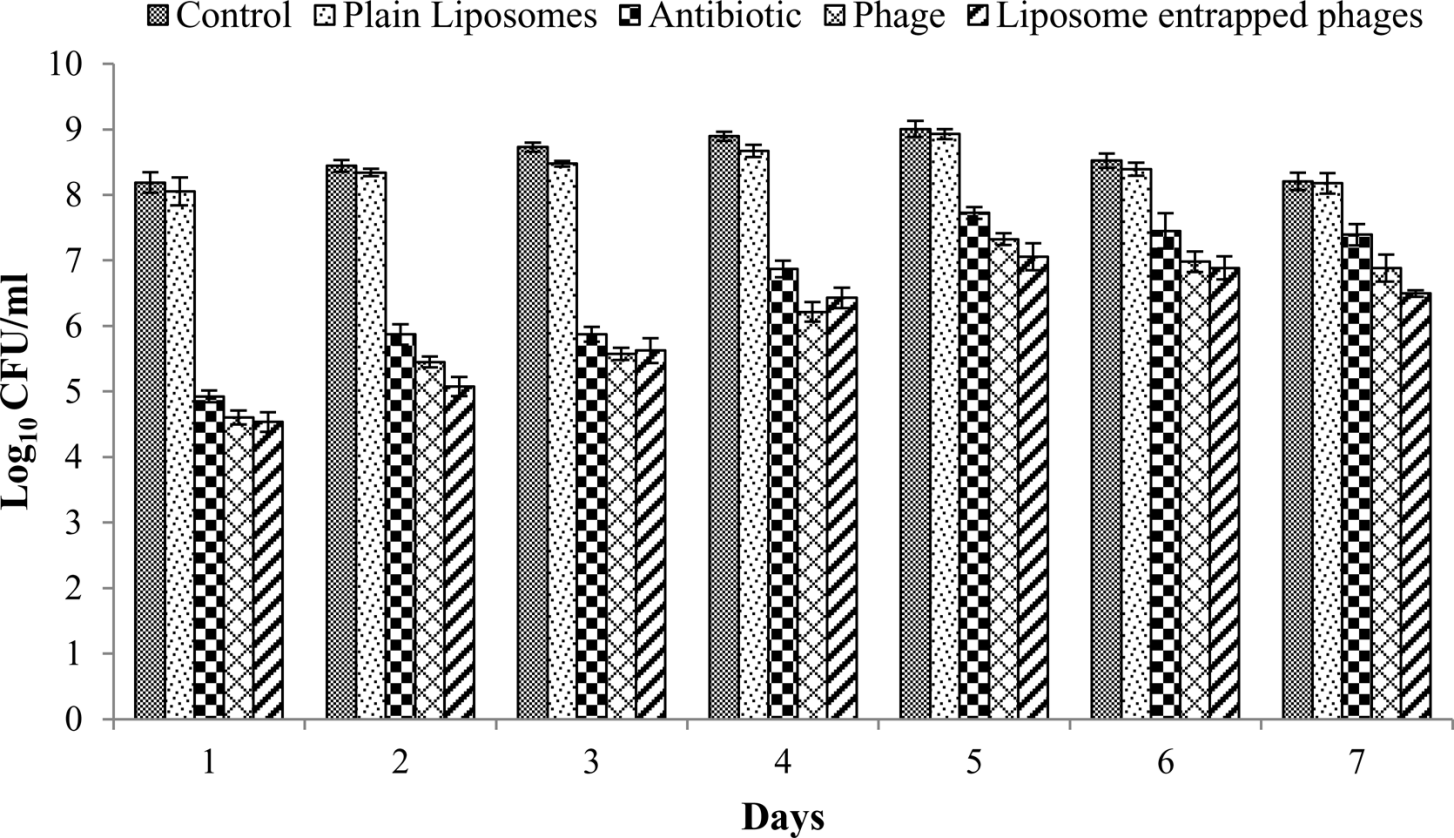
Bacterial count (CFU/ml) on different days of incubation of a 7 day biofilm of *K*. *pnuemoniae* B5055 in a microtiter plate following treatment with plain liposomes (empty lipoosmes as control), antibiotic (amikacin at 40 μg/ml), non-entrapped phages (MOI = 1) and liposome entrapped phages (MOI = 1). All values represent the mean ± SEM, calculated from two independent experiments, each performed in duplicate on different occasions.

Treatment of biofilm using liposome entrapped phage led to significant reduction of 3.7±0.1 logs in bacterial count up to day 4 of biofilm formation as compared to non-liposomal phage that caused reduction of 3.1±0.2 logs (Fig 2). From day 5 onwards, bacteriophage treatment of biofilm became insignificant (*P*>0.05) and reduction in log_10_ bacterial count fell to 1.3±0.2 logs. However, significant log reduction of 1.9±0.2 logs was observed when biofilm was treated with liposome entrapped phage. Treatment of biofilm using empty liposome (plain liposomes) did not cause any reduction in bacterial log count as compared to control, which confirmed that components of liposome had no biofilm eradicating potential.

#### Synergistic effect of phage and liposome entrapped phage with antibiotic

**Fig 3:**
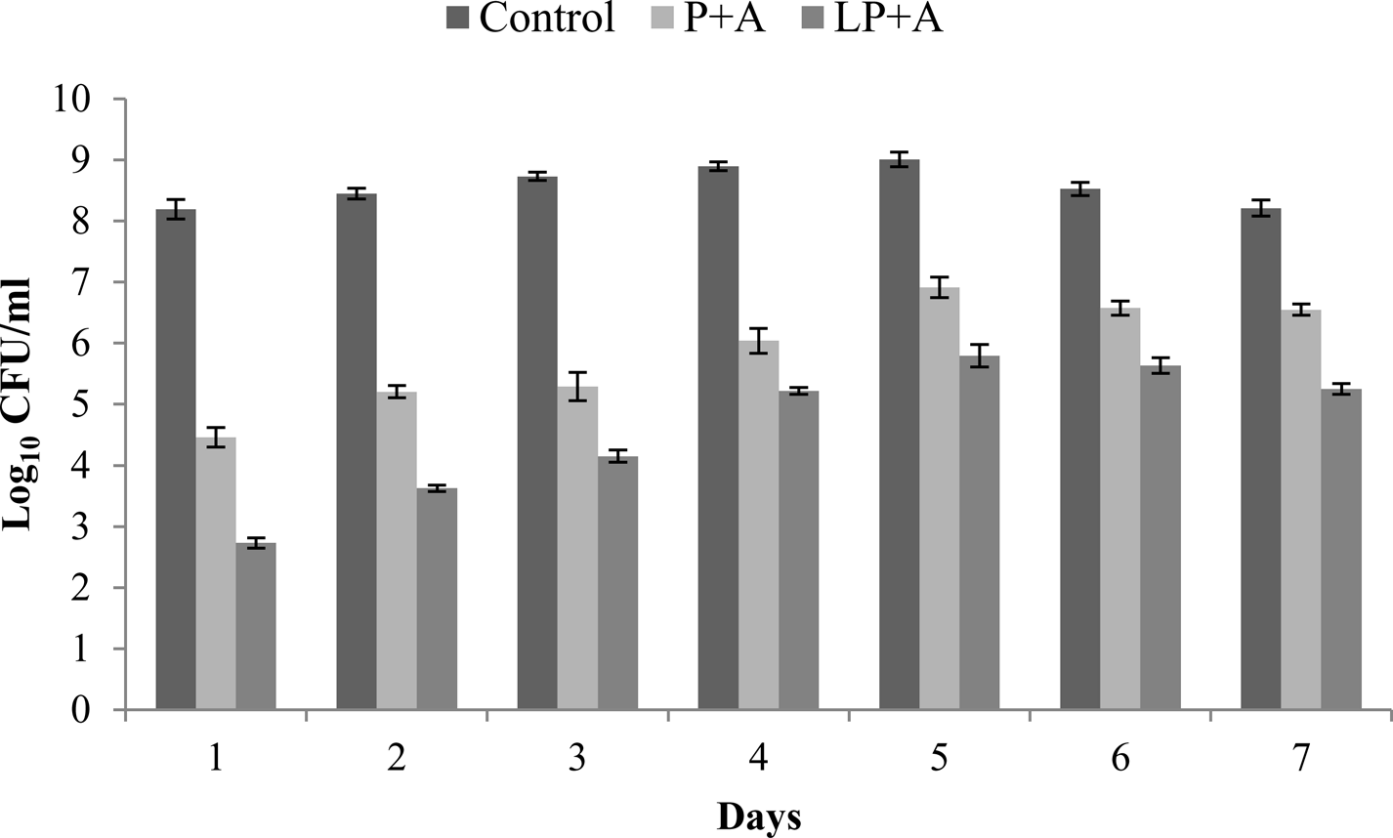
Bacterial count (CFU/ml) on different days of incubation of a 7 day biofilm of *K*.*pneumoniae* following treatment with phage + antibiotic (P+A) and liposome entrapped bacteriophage + antibiotic (LP+A) in a microtiter plate. All values represent the mean ± SEM, calculated from two independent experiments, each performed in duplicate on different occasions. A- Amikacin (40 μg/ml), P- Phage, LP- Liposome entrapped phage.

For younger biofilms (upto day 4 of biofilm), phage (MOI = 1) along with antibiotic (amikacin at 40 μg/ml) (P+A) caused reduction of 3.7±0.1 logs. On the other hand, significant reduction of 5.4±0.1 logs was observed when exposed to liposome entrapped phage (MOI = 1) with antibiotic (amikacin at 40 μg/ml) (LP+A). From day 5 onwards, P+A treatment of the biofilm became insignificant (*P*>0.05) and reduction in log bacterial count fell to 1.6±0.1 logs. Whereas significant (*P*<0.05) reduction of 3.21±0.2 logs was observed on treatment with LP+A (Fig 3). These results indicated that entrapped phage proved advantageous in eradicating the mature biofilm effectively especially when concentration of antibiotic was within the clinically achievable range.

### Phage neutralization test

Production of neutralizing antibodies is considered as one of the major hurdles in the clinical use of phage. So, keeping this in mind, antibodies against bacteriophage KPO1K2 were raised in mice and their neutralization effect on bacteriophage was checked by phage neutralization test. Phage alone (non-liposomal phage) as well as liposome entrapped phage formulation was tested for phage viability following interaction with antibodies (raised against phage) prepared in mice. It was observed that antibodies showed neutralizing ability for bacteriophage, even at low concentration as phage titer became zero after 3 h of incubation (Table 1). However, antibodies did not interfere with phage’s capacity to lyse susceptible bacteria following interaction with liposome entrapped phage even at a higher concentration. The phage titer of almost 5 logs remained constant and similar to that observed in control after 3 h of interaction.

**Table 1:** Comparison of effect of neutralizing antibodies on phage and liposome entrapped phage.

Treatment	Count (Log_10_ CFU or PFU/mL)				
	Phage			Bacteria	
	t_0_	t_3_	t_6_	t_3_	t_6_
Phage + antibodies (1X 10^−1^)	5.25 ± 0.25	ND	ND	5.08 ± 0.09	7.68 ± 0.21
Phage + antibodies (1X 10^−2^)	5.49 ± 0.31	ND	ND	4.94 ± 0.08	7.60 ± 0.18
Phage + normal serum	5.37 ± 0.28	5.32 ± 0.17	7.58 ± 0.29	4.97 ± 0.11	2.96 ± 0.23
Liposome entrapped phage + antibodies (1X 10^−1^)	5.30 ± 0.19	5.18 ± 0.12	7.13 ± 0.16	5.14 ± 0.15	2.82 ± 0.25

ND stands for not detectable

Treatment (**t**_**0**_**)** → estimate phage titer (**t**_**3**_**)** and add bacteria (**t**_**3**_**)** → estimate phage titer (**t**_**6**_**)** and bacterial count (**t**_**6**_**)** t_0,_ t_3,_ t_6_ in the table denotes sampling time of different treatments i.e. at 0 h, 3 h and 6 h

All values represent the mean ± SEM, calculated from two independent experiments, each performed in duplicate on different occasions.

To confirm the neutralization of phage, bacteria were added to antibody-coated phage particles after 3 h of incubation. A significant increase (*P*<0.05) of 2.6 logs in bacterial count upon subsequent incubation was observed due to neutralization of phage by antibodies. This resulted in no lysis of added bacteria. Whereas when bacteria were added in control tube (containing phage + normal mouse serum), as well as in tube containing liposomal formulation and antiserum (against phage) after 3 h, there was significant decrease (*P*<0.05) of 2 logs in bacterial count upon subsequent incubation of 3 h.

**Fig 4:**
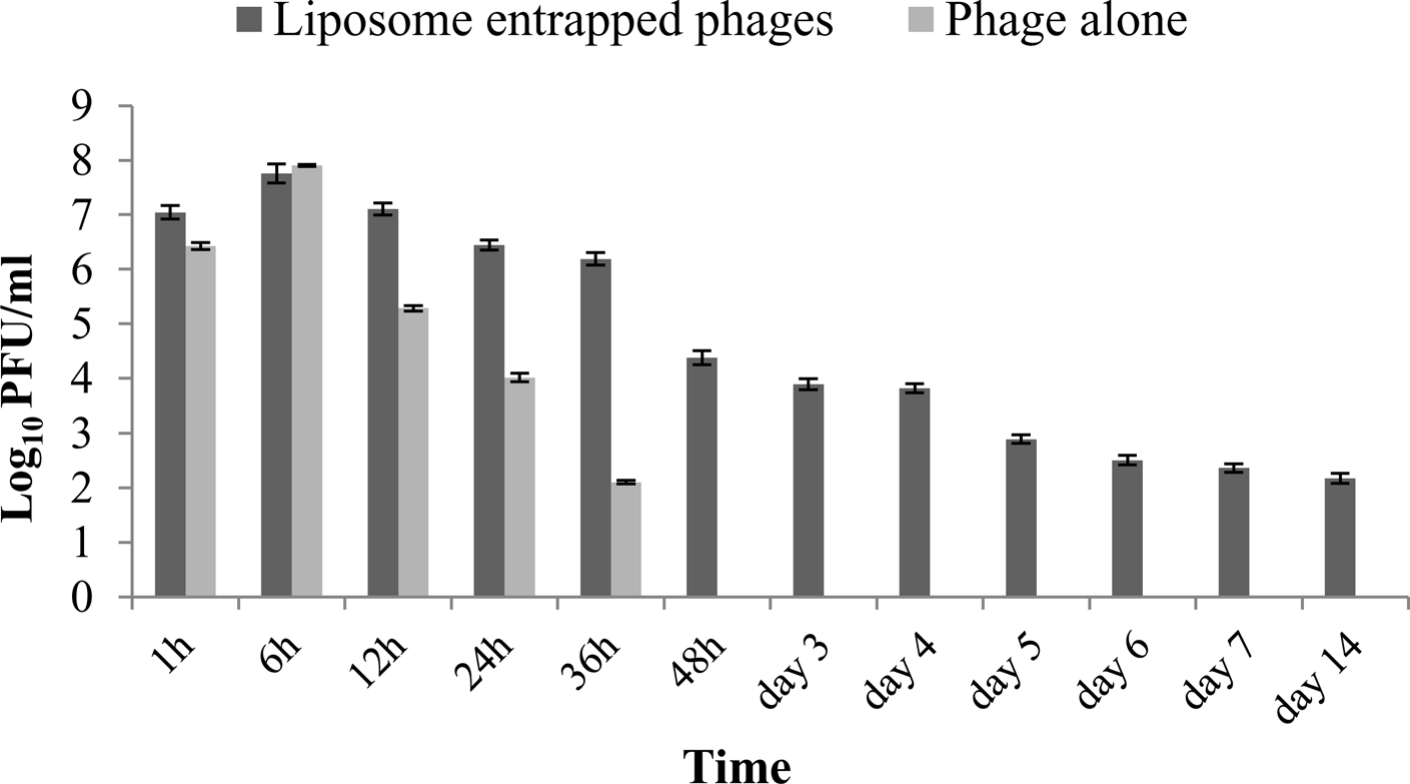
Biodistribution of liposome entrapped phage and unentrapped phage in spleen of BALB/c mice (n = 3). All values represent the mean ± SEM, calculated from two independent experiments, each performed in duplicate on different occasions

These results clearly showed that phage specific antibodies were able to react with free phage and neutralize it whereas phage entrapped in liposome remained protected from neutralizing antibodies as preincubation of liposome entrapped phage with antibodies did not inhibit the killing capacity of phage. This is clear from increasing number of PFU and decreasing number of CFU at 3 h. Thus liposome entrapped phage was able to persist inside the mouse system even in presence of antibodies to phage. This characteristic of liposome in providing protection to phage from neutralizing antibodies is biologically relevant and demonstrates how delivery system can improve the survival of phage *in vivo* (Fig 4).

### Phagocytic uptake and killing

**Fig 5:**
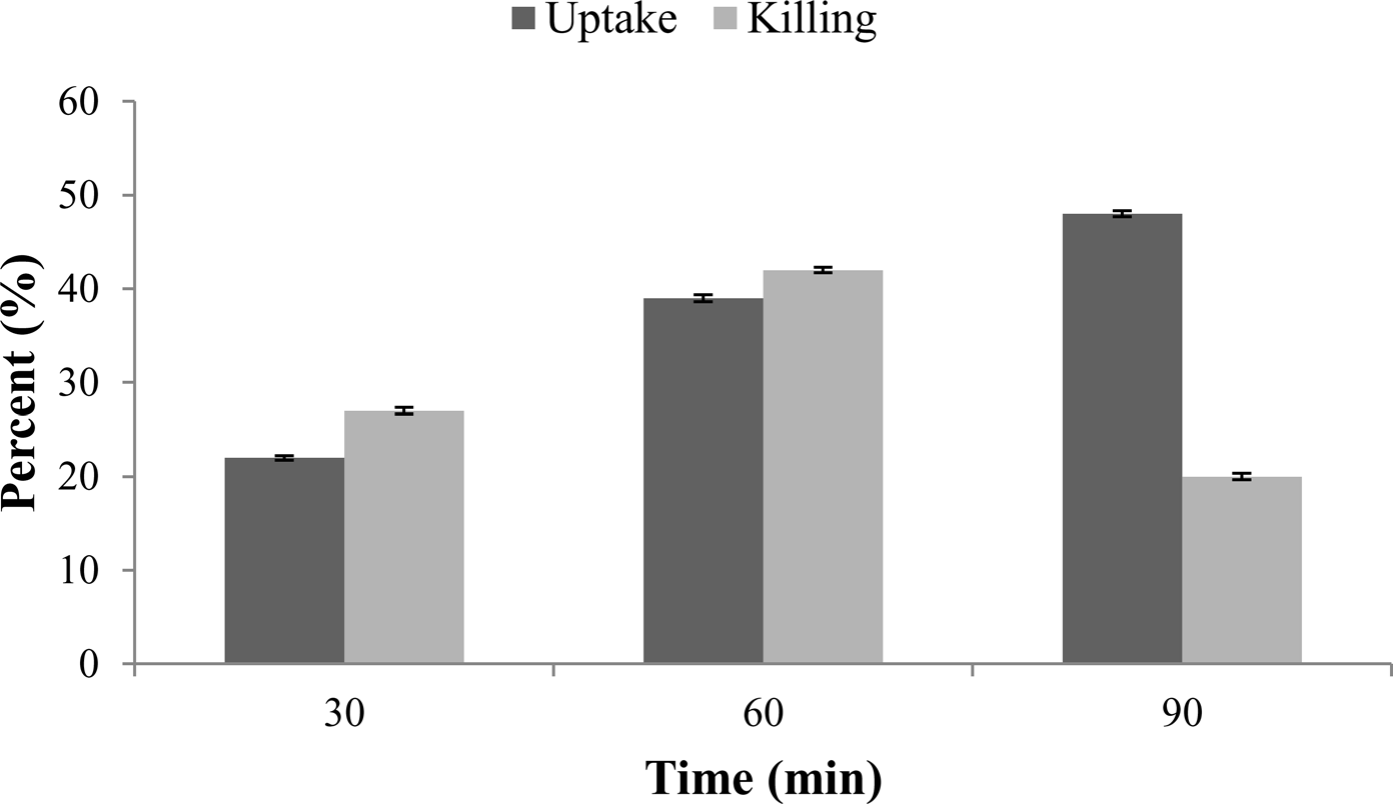
Percent bacterial uptake and killing by mouse peritoneal macrophages at different time intervals. All values represent the mean ± SEM, calculated from two independent experiments, each performed in duplicate on different occasions. SEM represent the 95% confidence interval. Confidence interval is calculated as SEM * 3.18. Raw data is available in Table 2.

The results of bacterial uptake and killing are presented in Fig 5. Supporting data (S1 Table) presents the raw data of Fig 5. Within 30 min of interaction of *K*. *pneumoniae* B5055 with mouse peritoneal macrophages, a bacterial uptake of ~22% was observed that increased to 39% at 60 min and 48% at 90 min. A bacterial kill of ~27% was observed at 30 min and killing rate increased to ~42% at 60 min and then decreased to 20% by 90 min. All values were calculated from two independent experiments, each performed in duplicate on different occasions. To obtain a confidence interval, we multiplied S_n_ (SEM) by a t value (obtained from a table of ***t*** values), using the df (degree of freedom) in our original data. For example, the 95% confidence interval for 22 ± 0.25 would be 22 ± 0.25 * 3.18. So,

Lower confidence limit (LCL) = 21.21

Upper confidence limit (UCL) = 22.80

### Comparison of intracellular killing activity of phage and liposome entrapped phage at different MOI

The killing of phagocytised bacteria following treatment with phage or liposome entrapped phage was assessed at different MOI. After 1.5 h of phagocytic uptake of *K*. *pneumoniae*, phage or liposome entrapped phage was added at different MOI. Liposome entrapped phage showed significantly higher bactericidal activity (94.6% killing of phagocytised bacteria) than that observed with phage (non-liposomal phage) treatment, throughout the period of observation (*P*<0.05). The results in Table 2 show that about 21.4% of intracellular bacteria present in macrophages were killed after 24 h when macrophages with phagocytised bacteria were exposed to phage at 100 MOI. However, when liposome entrapped phage was taken up by macrophages, 94.6% killing of intracellular bacteria was achieved even at 10 MOI after 24 h (Table 2).

**Table 2:** Comparison of percent killing of intracellular bacteria when treated with phage and liposome entrapped phage.

Time after exposure	Percent killing of bacteria (%)			
	Phage		Liposome entrapped phage	
	MOI = 10	MOI = 100	MOI = 10	MOI = 100
3h	2.92 ± 1.69	4.09 ± 0.73	28.65 ± 2.35	30.40 ± 2.04
12h	10.95 ± 2.67	19.30 ± 1.14	45.24 ± 0.92	46.10 ± 1.30
24h	15.38 ± 2.13	21.36 ± 1.62	94.6 ± 15.65	94.6 ± 16.57

All values represent the mean ± SEM, calculated from two independent experiments, each performed in duplicate on different occasions. SEM represent the 95% confidence interval. Confidence interval is calculated as SEM * 3.18

**Fig 6:**
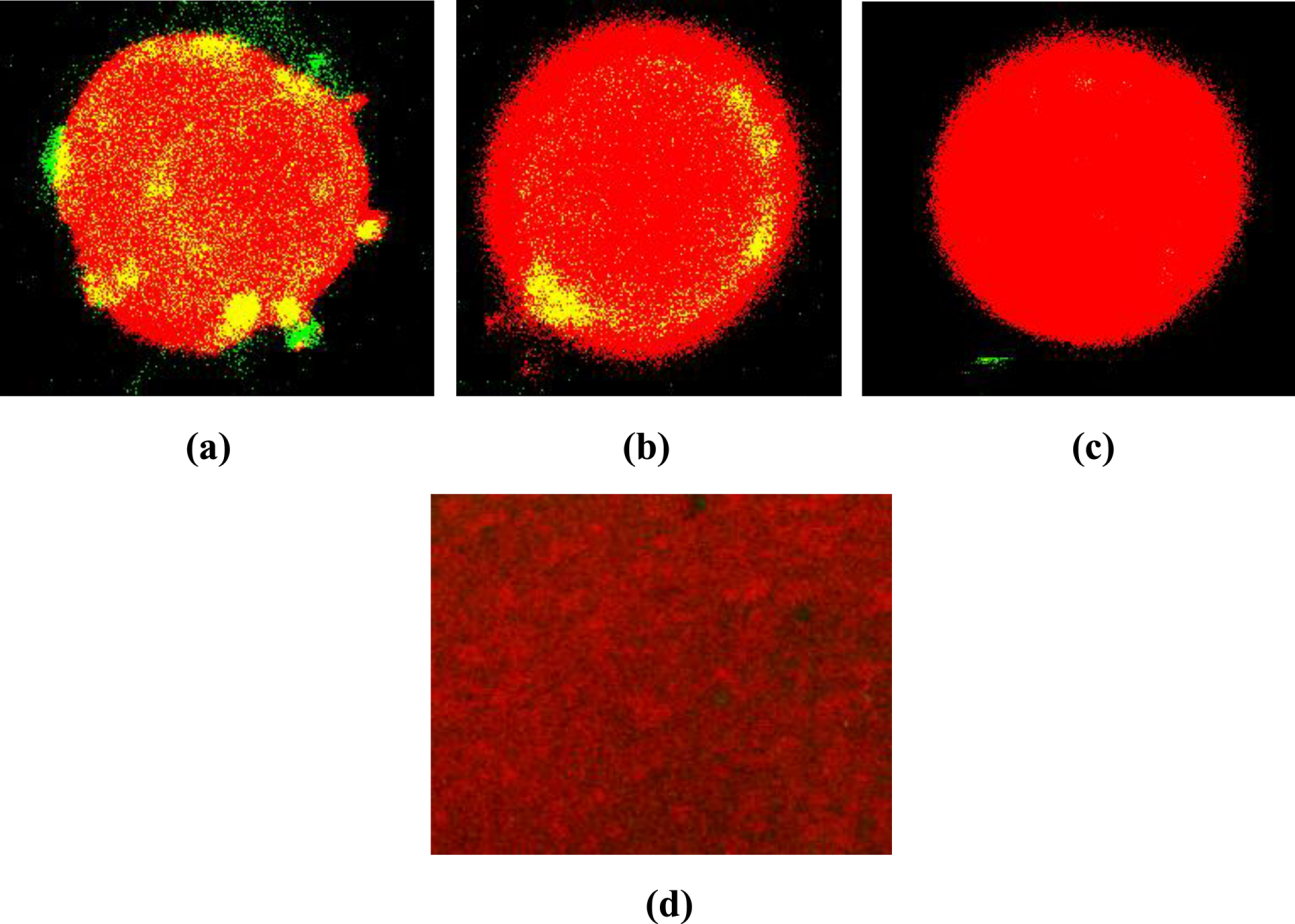
Visualization of a representative field of a) untreated macrophages having intracellular *K*. *pneumoniae* b) infected macrophages treated with bacteriophage at a MOI of 10 c) infected macrophages treated with liposome entrapped bacteriophage at a MOI of 10. d) control slide of heat killed bacteria stained with Live/Dead bacterial stain under confocal microscope (at 60X). The live bacteria were stained green and dead bacteria stained red.

Confocal microscopy of macrophages stained with Live/dead staining kit also confirmed the difference in the killing activity of phage and liposome entrapped phage (Fig 6). Both green (live cells) and red fluorescing (dead cells) intracellular bacteria were observed inside macrophages when treated *in-vitro* with phage whereas only red fluorescing cells were observed following treatment with liposome entrapped bacteriophage (Fig 6). The ratio of live/dead cells in control well was 11.7 (Fig 6A). The ratio of live/dead cells decreased to 3.7 in phage treated well (Fig 6B). There was significant decrease (*P<0*.*05*) in the ratio of live/dead cells in liposomal phage treated well to 0.05 (Fig 6C).

## Discussion

Treatment of *K*. *pneumoniae* mediated infections with antibiotics is becoming increasingly difficult due to the widespread prevalence of multi-drug resistant strains. Bacteriophage therapy that includes use of lytic phage or their products or both, represents an alternative tool in the treatment of *K*. *pneumoniae* infections, especially those that are refractory to the action of antibiotics. The efficacy of bacteriophage in clearing *K*. *pneumoniae* infection in animal models has been established. Inspite of these encouraging results the role of neutralising antibodies and effect of phage on intracellular bacteria due to their restricted intracellular entry remains to be investigated. Moreover increasing problem of antibiotic resistance coupled with inability to eradicate the biofilm structures has increased the demand of novel strategies for biofilm eradication. In order to overcome these hurdles the present study was conducted by employing cationic liposomes as delivery vehicle for phage particles.

Bacteriophages have been suggested as effective antibiofilm agents [24,25]. In the present study, efficacy of phage and liposome entrapped phage, alone and in conjunction with antibiotic was employed for eradicating the biofilm. The results showed that young biofilm was eradicated by using either of the two systems independently. Phage alone on the contrary was unable to eradicate the mature biofilm, whereas liposomal formulation of phage could curb its formation *in vitro*. This might be due to the inaccessibility of deeper layers of bacteria to phage in a biofilm as progeny phage released from lysed bacteria diffuses radially (convection transport) and not through downstream flow (advective transport) [26]. These results are consistent with the observation made by Doolittle *et al*. [26] who reported full infection of thin *Escherichia coli* biofilm by its specific phage compared to thicker biofilm of *Pseudomonas aeruginosa*, that was only infected at the surface and access to deeper layers was restricted. Liposomes as drug carriers are very promising in preventing biofilm formation and treatment [27,28]. The main aim of liposomal drug application is to overcome two major obstacles, penetration of drug into the target matrix so as to reach biofilm bacteria and release of drug in the vicinity of microorganisms. This approach has significantly increased the local drug concentration and simplified targeted delivery. The results of the present study showed that phage entrapped in cationic liposome was able to penetrate the interior of biofilm matrix leading to eradication of mature biofilm. This observation is in confirmation with the results of Meers *et al*. [29] who also showed that amikacin containing liposomes could effectively penetrate inside *Pseudomonas* biofilm resulting in enhanced concentration of antibiotic within the biofilm. It has also been demonstrated that positively and negatively charged liposomes have the ability to penetrate inside the biofilm formed by oral and skin bacteria [29,30].

Cornelissen *et al*. [31] showed that degradation of older biofilm using bacteriophage is more difficult as compared to young biofilm. As biofilm ages, phage resistant mutants are likely to develop within the biofilm layers [32]. Beaulac *et al*. [33] on the other hand showed that liposome-encapsulated antibiotic not only increased the efficacy of antibiotic treatment of non-resistant strains but also helped in overcoming bacterial resistance. The results of this study also confirm increased susceptibility of mature biofilm to combined treatment of antibiotic and liposome entrapped phage as compared to un-entrapped phage with or without antibiotic. It might prove to be a good strategy as combined phage therapy with antibiotics can help in preventing re-establishment of biofilm by phage resistant mutants. The increased antimicrobial efficacy in this case is not correlated with rapid drug release from liposomal formulation, but rather it probably is due to electrostatic interaction/fusion between liposome and bacterial cell. Cationic vesicles interact more readily with negatively charged outer membrane of gram-negative bacteria than do anionic and neutral liposomes. This is in agreement with the results of other studies where fluid cationic liposomes have been shown to prevent biofilm formation by *Staphylococci*, *Pseudomonas aeruginosa* and oral bacteria [28]. Therefore, cationic liposomes used in the present study are good candidate for the delivery of bacteriophage to mature biofilm of *K*. *pneumoniae*.

A number of concerns have been raised on the therapeutic use of phage *in vivo*. The foremost issue has been the mammalian host immune response that theoretically will render the phage inactive upon repeated dosing due to the formation of neutralizing antibodies. To prove this point, antibody against bacteriophage KPO1K2 was raised in mice. The results of present study showed that bacteriophage got inactivated within 3 h of incubation of phage with antiserum containing phage specific antibodies as no phage particles were detected thereafter, suggesting that antibodies against phage were able to neutralize the phage. The liposomal membrane provided almost 100% protection to phage from neutralizing antibodies as phage titre of liposomal entrapped phage remained constant after 3 h of exposure to antibodies. When bacteria were added after 3 h to the tube containing phage and antibodies, significant increase (*P*<0.05) in bacterial log_10_ count was observed following extended incubation. These results confirmed that no phage was present in the preparation. Whereas addition of bacteria to the tube containing liposomal phage and antibodies caused significant (*P*<0.05) log_10_ reduction in bacterial count confirming that phage inside liposome was protected from neutralizing antibodies.

Previous researchers have suggested that bacteriophage can be used to treat bacterial infections as long as bacteria remain accessible and hence, phage therapy is not suitable for intracellular bacteria. Though *K*. *pneumoniae* is considered as an extracellular pathogen but it can survive inside infected mouse macrophages *in vivo*. Tullio *et al*. [34] demonstrated that phagocytised *Klebsiella* remained viable in macrophages even after 24 h indicating intracellular survival of bacteria. Working in this direction, killing capacity of phage and liposome entrapped phage against bacteria present within macrophages was compared. Since phage on its own is not able to enter the macrophage hence, free phage added to the infected macrophages did not cause significant reduction (*P*>0.05) in the number of intracellular bacteria. This is in agreement with the results of Capparelli *et al*. [23] who also reported that phage particles were unable to show bactericidal effect against *S*. *aureus* inside macrophages. On the contrary, liposome entrapped phage in this study caused 94.6% killing of intracellular bacteria present inside macrophages within 24 h. Analysis of live/dead stain results using ImageJ software also confirmed that liposomal phage caused significant decrease in live/dead cells ratio (0.05) as compared to control and phage alone. Other researchers have also reported enhanced killing of intracellular microorganisms such as *Staphylococcus aureus* [35], *Escherichia coli*, *Brucella abortus*, *Brucella canis* and *Mycobacterium avium* complex (MAC) as phagocytosis of aminoglycoside-loaded liposomes yielded higher intracellular concentration of therapeutic drug [36–41]. These results suggest that this is due to the ability of liposome to release its entrapped contents, phage or antibiotic following entry inside macrophage that finally makes an impact on the growth of intracellular bacteria, eliminating them completely.

In earlier studies the inability of phage to kill intracellular bacteria and production of neutralizing antibodies has been considered a major drawback of phage therapy. For the first time results of this study conclude that liposomal membrane provides protection to phage from neutralizing antibodies and helps the phage to enter into phagocytic cells and kill already engulfed *K*. *pneumoniae* present within macrophages. In addition, liposome entrapped phage also showed synergistic activity along with amikacin to eradicate mature biofilm of *K*. *pneumoniae*. This approach shall not only restrict intracellular proliferation of bacteria within the phagocytic cells but also protect the host from further relapse of infection and treatment failures.

## Supporting Information

S1 Table Percent bacterial uptake and killing by mouse peritoneal macrophages at different time intervals. All values represent the mean ± SEM, calculated from two independent experiments, each performed in duplicate on different occasions. SEM represent the 95% confidence interval. Confidence interval is calculated as SEM * 3.18.
(DOCX)
