# Supplementary material for: Encapsulation of Bacteriophage in Liposome Accentuates Its Entry in to Macrophage and Shields It from Neutralizing Antibodies
Source: PLoS One. 2016 Apr 26;11(4):e0153777. doi: 10.1371/journal.pone.0153777 (PMC4846161; doi:10.1371/journal.pone.0153777)
Supplement: S1 Table — All values represent the mean ± SEM, calculated from two independent experiments, each performed in duplicate on different occasions. SEM represent the 95% confidence interval. Confidence interval is calculated as SEM * 3.18. (DOCX) [file pone.0153777.s001.docx]

**S1 Table.**

| Time (in minutes) | % Uptake of bacteria | % Killing of intracellular bacteria |
| --- | --- | --- |
| 30 | 22±0.80 | 27±1.14 |
| 60 | 39±1.21 | 42±0.92 |
| 90 | 48±0.99 | 20±1.11 |

Note: % killing of intracellular bacteria was calculated out of % uptake of bacteria at the particular time period (formula given in the manuscript).
